# Association between triglyceride-glucose body mass index and risk of hypertensive disorders of pregnancy: a case-control study

**DOI:** 10.3389/fmed.2026.1844562

**Published:** 2026-06-25

**Authors:** Jun Liu, Haoran Ma, Xi Chen, Xueyun Chang

**Affiliations:** 1 Department of Obstetrics, Fuyang Women's and Children's Hospital, Fuyang, Anhui, China; 2 Department of Respiratory and Critical Care Medicine, Fuyang Women's and Children's Hospital, Fuyang, Anhui, China; 3 Department of Ultrasound, Fuyang Women's and Children's Hospital, Fuyang, Anhui, China

**Keywords:** case-control study, hypertensive disorders of pregnancy, insulin resistance, obesity, triglyceride-glucose body mass index

## Abstract

**Objective:**

To investigate the association between triglyceride-glucose body mass index (TyG-BMI) and the risk of hypertensive disorders of pregnancy (HDP), and to compare the discriminative ability of TyG-BMI with triglyceride-glucose index (TyG) and body mass index (BMI) for HDP.

**Methods:**

A case-control study was conducted including 346 pregnant women hospitalized in the Department of Obstetrics at Fuyang Women and Children's Hospital from January 2021 to December 2025. Participants were divided into HDP group (*n* = 168) and control group (*n* = 178) based on HDP diagnosis. General clinical data, physical examinations, and hematological parameters at admission were collected for both groups. BMI, TyG index, and TyG-BMI were calculated. Multivariable logistic regression was used to analyze the independent association between TyG-BMI and HDP. Restricted cubic spline (RCS) curves were employed to explore dose-response relationships. Receiver operating characteristic (ROC) curves were used to compare the discriminative performance of different indices.

**Results:**

The TyG-BMI level in the HDP group was significantly higher than that in the control group (228.4 ± 44.6 vs. 196.8 ± 36.2, *P* < 0.001). Multivariable logistic regression analysis showed that after adjusting for confounding factors, each 10-unit increase in TyG-BMI was associated with a 30% increase in HDP risk (adjusted OR = 1.30, 95%CI: 1.16–1.46, *P* < 0.001). RCS analysis demonstrated a non-linear positive association between TyG-BMI and HDP risk (*P* for non-linearity = 0.026). ROC analysis revealed that the area under the curve (AUC) for TyG-BMI in discriminating HDP was 0.782 (95%CI: 0.734–0.830), which was significantly superior to TyG index (AUC = 0.708, *P* = 0.005) and BMI (AUC = 0.694, *P* < 0.001). Subgroup analysis showed that the association between TyG-BMI and HDP remained consistent across all subgroups (all *P* for interaction > 0.05).

**Conclusion:**

TyG-BMI is independently and positively associated with HDP, and its discriminative performance is superior to TyG index and BMI. It can serve as a comprehensive indicator for identifying and assessing the degree of metabolic abnormalities in HDP patients.

## 1 Introduction

HDP represent serious pregnancy-specific complications encompassing various clinical types including gestational hypertension, preeclampsia, eclampsia, and chronic hypertension with superimposed preeclampsia. The global incidence ranges from 5% to 10%, reaching 10% to 15% in developing countries (1). A multicenter cross-sectional study based on hospital data in China reported that the incidence of HDP among hospitalized pregnant women ranged from 5.6% to 9.4%, with an increasing trend in recent years (2). HDP is the second leading cause of maternal mortality worldwide, accounting for approximately 14% of all maternal deaths, and is also a significant factor contributing to perinatal mortality, preterm birth, and fetal growth restriction (3). Moreover, HDP not only causes acute adverse effects on mothers and infants but is also closely associated with increased risks of long-term cardiovascular disease, chronic kidney disease, and type 2 diabetes mellitus in mothers, with these elevated risks persisting for decades postpartum (4). Therefore, in-depth exploration of metabolic risk factors and their characteristics in HDP is of significant clinical importance for disease risk assessment.

The pathogenesis of HDP remains incompletely understood. The currently accepted “two-stage” theory proposes that the first stage involves inadequate trophoblast invasion during early pregnancy, leading to impaired spiral artery remodeling and shallow placental implantation, while the second stage involves systemic vascular endothelial dysfunction and inflammatory responses secondary to placental ischemia and hypoxia (5). In this pathological process, accumulating evidence indicates that insulin resistance plays a crucial role. Normal pregnancy itself involves physiological IR to ensure adequate glucose supply to the fetus. However, when physiological IR is excessively amplified to pathological IR, it can significantly increase HDP risk through multiple mechanisms including inducing compensatory hyperinsulinemia, activating the sympathetic nervous system, promoting renal tubular sodium reabsorption, and impairing nitric oxide-mediated vascular endothelial relaxation function (6). A meta-analysis including 12 prospective studies showed that pregnant women with IR levels in the highest quartile had approximately 2.6 times higher risk of developing preeclampsia compared to those in the lowest quartile (7). Similarly, obesity, as an important driver and independent risk factor for IR, is closely associated with increased HDP risk. Under obese conditions, increased secretion of pro-inflammatory cytokines (such as tumor necrosis factor-α and interleukin-6) from adipose tissue and decreased protective adipokines (such as adiponectin) can further exacerbate IR, oxidative stress, and vascular endothelial damage, creating a vicious cycle (8). The large-scale cohort study by Cnattingius et al. (9) demonstrated that each 5 kg/m^2^ increase in body mass index (BMI) was associated with approximately 1.5–2.0 times higher preeclampsia risk. Among the above research evidence, part of the metabolic regulatory mechanism conclusions are derived from general non-pregnant cardiometabolic population studies, which can serve as indirect theoretical basis for explaining metabolic disorder pathways; while the cohort data and meta-analysis results focusing on pregnant women are direct pregnancy-specific evidence confirming the causal association between IR, obesity and HDP occurrence.This evidence fully demonstrates that IR and obesity are two core and interrelated metabolic dimensions in HDP pathogenesis.

However, accurate assessment of IR in clinical practice typically relies on hyperinsulinemic-euglycemic clamp tests or homeostatic model assessment of insulin resistance (HOMA-IR). The former is operationally complex and costly, making it difficult to implement in routine clinical practice, while the latter requires measurement of fasting insulin levels, which is not a routine test in many primary and secondary healthcare institutions. Additionally, the standardization and inter-laboratory consistency of insulin assays remain insufficient (10). In this context, the TyG, a simple IR surrogate indicator requiring only fasting triglycerides (TG) and fasting plasma glucose (FPG) for calculation, has gained widespread attention. Simental-Mendía et al. (11) confirmed that the TyG index showed good correlation with IR assessed by hyperinsulinemic-euglycemic clamp tests (*r* = 0.68, *P* < 0.001) and demonstrated reliable IR discriminative ability in the general population and various metabolic diseases. Notably, this verification conclusion of TyG index is mainly based on non-pregnant adult populations, and its diagnostic stability and optimal cutoff value in pregnant women cannot be directly generalized; recent obstetric targeted studies have confirmed that single TyG index has obvious application limitations in gestational metabolic risk evaluation (12).Nevertheless, the TyG index only reflects IR status at the glycolipid metabolic level and lacks assessment of the obesity dimension. While BMI is the most commonly used indicator for assessing overall obesity, it cannot reflect the intrinsic degree of metabolic abnormalities. Previous studies have indicated that single-dimensional assessment may miss risk information from phenotypic groups such as “metabolically obese normal weight” or “metabolically healthy obesity,” thereby reducing disease discriminative efficacy (13).

To overcome the limitations of single indicators, Er et al. (14) proposed the TyG-BMI in 2016, a novel composite metabolic indicator calculated as the product of TyG index and BMI, mathematically achieving simultaneous integration of information from both IR and obesity dimensions. Their study in Korean non-diabetic populations confirmed that TyG-BMI's efficacy in discriminating IR was significantly superior to TyG index and other TyG-derived indicators (TyG-waist circumference, TyG-waist-to-hip ratio). Subsequently, the application value of TyG-BMI in various metabolism-related diseases has been extensively validated. In the cardiovascular field, Xia et al. (15) reported that TyG-BMI was significantly positively associated with cardiovascular event incidence and demonstrated superior predictive efficacy compared to traditional lipid metabolism indicators. Additionally, TyG-BMI has shown superior performance in discriminating non-alcoholic fatty liver disease (16) and metabolic syndrome (17).The above application researches are mostly carried out in non-pregnant chronic disease populations, which can provide indirect methodological reference for composite metabolic indicators, but cannot clarify its clinical application effect in obstetric scenarios. These studies suggest that TyG-BMI, by integrating synergistic effect information of IR and obesity, can more comprehensively reflect individual metabolic risk status compared to its individual components.

Currently, research on the association between TyG-BMI and HDP remains scarce. Existing pregnancy-related studies have mostly focused on individual associations of TyG index or BMI with HDP. Zheng et al. (18) reported in a retrospective cohort that elevated TyG index during pregnancy was associated with increased preeclampsia risk but did not include TyG-BMI for comparative analysis. Whether TyG-BMI, as a composite indicator integrating dual metabolic information of IR and obesity, can better reflect metabolic characteristics of HDP patients and demonstrate superior discriminative efficacy compared to its component indicators lacks systematic research evidence. Furthermore, existing studies are mostly based on early pregnancy or pre-pregnancy data, while in clinical practice, a considerable proportion of HDP patients are first hospitalized for diagnosis and treatment in the second or third trimester due to elevated blood pressure or related symptoms. Clinical data at admission represents the most complete and standardized data source. Understanding how to utilize routine examination data at admission to assess the association between TyG-BMI and HDP has important practical application value.

In summary, this study aims to systematically investigate the association between TyG-BMI and HDP and its dose-response relationship based on clinical data from pregnant women hospitalized in the Department of Obstetrics at Fuyang Women and Children's Hospital, using a case-control study design. We also directly compare the discriminative efficacy differences between TyG-BMI and TyG index, BMI for HDP, with the goal of providing new scientific evidence for in-depth understanding of metabolic characteristics of HDP and offering a simple, economical composite indicator for clinical assessment of metabolic risk in HDP patients.

## 2 Materials and methods

### 2.1 Study design and participants

This single-center retrospective case-control study enrolled hospitalized pregnant women at the Obstetrics Department of Fuyang Women and Children's Hospital between January 2021 and December 2025, with clinical data extracted from electronic medical records.Inclusion criteria for both groups included singleton pregnancy, age 18–45 years and complete admission clinical and laboratory data. Patients in the HDP group were diagnosed with hypertensive disorders of pregnancy, while controls were normotensive pregnant women admitted for routine prenatal care or delivery without gestational hypertension-related diseases. Common exclusion criteria covered pregestational chronic metabolic, renal, autoimmune and uncontrolled thyroid diseases, multiple pregnancy, assisted reproductive conception, use of glycolipid metabolism-altering drugs, severe hepatorenal dysfunction, gestational diabetes mellitus and incomplete core data. In total, 346 participants were finally enrolled, consisting of 168 HDP cases and 178 healthy controls. This study was approved by the hospital ethics committee and complied with the Declaration of Helsinki. Informed consent was exempted due to its retrospective design.

### 2.2 Sample size calculation

Based on preliminary experimental data, the control group TyG-BMI mean was approximately 197 with standard deviation of 36, while the HDP group TyG-BMI mean was approximately 228 with standard deviation of 45. Setting α = 0.05, β = 0.10 (test power 1–β = 0.90), and approximately 1:1 allocation ratio between groups, using the sample size calculation formula for comparing means between two independent samples, the minimum required sample size was 140 per group, totaling 280 cases. Considering approximately 15% data missing rate, at least 322 cases needed to be included. This study ultimately included 346 cases, meeting statistical requirements.

### 2.3 HDP diagnostic criteria

HDP diagnoses were established in accordance with the American College of Obstetricians and Gynecologists (ACOG) criteria (19).Gestational hypertension was defined as new-onset elevated blood pressure after 20 weeks of gestation without proteinuria. Preeclampsia referred to gestational hypertension combined with proteinuria or multi-organ impairment. Severe preeclampsia presented with markedly elevated blood pressure or serious complications, and eclampsia was defined as preeclampsia complicated by unexplained convulsions. All diagnoses were verified by senior obstetricians. All HDP subtypes were merged for primary analysis, and subtype-stratified analyses were further performed in sensitivity analyses.

### 2.4 Data collection

Clinical data were collected from electronic medical records, including age, gestational age at admission, gravidity, parity, and previous adverse pregnancy history. At admission, trained nurses measured height and weight (to calculate BMI) and seated resting blood pressure (average of three consecutive measurements); for HDP patients, pre-treatment admission blood pressure was recorded, and for controls, admission blood pressure was documented. All participants underwent post-admission laboratory tests, including fasting plasma glucose (FPG), triglycerides (TG), total cholesterol (TC), high-density lipoprotein cholesterol (HDL-C), low-density lipoprotein cholesterol (LDL-C), alanine aminotransferase (ALT), aspartate aminotransferase (AST), serum creatinine (Scr), blood urea nitrogen (BUN), albumin (ALB), hemoglobin (Hb), and platelet count (PLT). For the HDP group, laboratory tests were performed on pre-treatment admission blood samples to reflect baseline metabolic status.

### 2.5 Index calculations

This study used height and weight measured at admission to calculate BMI using the formula BMI (kg/m^2^) = weight (kg)/height^2^ (m^2^) to reflect body composition status at admission. The TyG was calculated based on fasting triglyceride and fasting plasma glucose levels using the formula TyG = Ln [TG (mg/dL) × FPG (mg/dL)/2], where the conversion factors were 1 mmol/L = 88.57 mg/dL for TG and 1 mmol/L = 18.02 mg/dL for FPG. The TyG-BMI was obtained by multiplying the TyG index by BMI, i.e., TyG-BMI = TyG × BMI (kg/m^2^), thus integrating information from both insulin resistance and obesity dimensions into a single indicator.

### 2.6 Statistical analysis

Statistical analyses were performed using R software (version 4.3.1) and SPSS 26.0. Normally distributed continuous variables were expressed as mean ± standard deviation and compared between groups using independent samples *t*-test. Non-normally distributed continuous variables were expressed as median (interquartile range) [M (IQR)] and compared between groups using Mann-Whitney *U*-test. Categorical variables were expressed as frequency (percentage) [*n* (%)] and compared between groups using χ^2^ test. Normality testing was performed using the Shapiro-Wilk test.

Spearman correlation analysis was used to assess correlations between TyG-BMI and other variables. Multivariable logistic regression analysis was used to assess associations between TyG-BMI, TyG index, BMI, and HDP, with results expressed as odds ratios (OR) and 95% confidence intervals (CI). The following models were established: Model 1 (unadjusted): Univariate analysis. Model 2: Adjusted for age, gestational age at admission, and gravidity/parity. Model 3 (fully adjusted model): Further adjusted for TC, HDL-C, UA, and previous adverse pregnancy history based on Model 2.To avoid multicollinearity, component variables of TyG-BMI (TG, FPG, BMI) were not included as covariates. SBP and DBP, as components of HDP diagnostic criteria, have high intrinsic association with the outcome variable (over-adjustment bias) and were not included as covariates. When analyzing TyG index association with HDP, BMI was not included as a covariate; when analyzing BMI association with HDP, TG and FPG were not included as covariates. Multicollinearity of other variables was assessed using variance inflation factor (VIF).

TyG-BMI was categorized into quartiles (Q1–Q4), with Q1 as the reference group, calculating OR values and 95%CI for each group, with trend testing performed. Similar quartile analyses were conducted for TyG index and BMI.RCS functions were used to explore potential non-linear dose-response relationships between TyG-BMI, TyG index, BMI, and HDP risk, with knots set at the 5th, 25th, 50th, 75th, and 95th percentiles, using the median as the reference point.The following subgroup analyses were conducted to assess result robustness: age (< 30 years/≥30 years), BMI (< 28/≥28 kg/m^2^), gravidity/parity (nulliparous/multiparous), and gestational age at admission (< 37 weeks/≥37 weeks), with interaction effects tested.Receiver operating characteristic (ROC) curves were used to assess the efficacy of TyG-BMI, TyG index, and BMI for discriminating HDP, with AUC compared using DeLong test.Sensitivity, specificity, Youden index, positive likelihood ratio (+LR), and negative likelihood ratio (–LR) were calculated to determine optimal cutoff values. Model goodness of fit was assessed using the Hosmer-Lemeshow test.The following sensitivity analyses were conducted to verify result robustness: (1) excluding extreme values of upper and lower 1% of TyG-BMI; (2) excluding patients with severe preeclampsia; (3) stratified analysis by HDP subtype; (4) sensitivity analysis after adjusting for gestational age at admission; (5) propensity score matching analysis; (6) excluding pregnant women with BMI ≥ 30 kg/m^2^; (7) excluding pregnant women with FPG ≥ 5.1 mmol/L. All tests were two-sided, with *P* < 0.05 considered statistically significant.

## 3 Results

### 3.1 Baseline characteristics of the study population

A total of 346 pregnant women were enrolled, with a mean age of 29.6 ± 4.6 years. The cohort included 168 HDP patients (48.6%), consisting of 96 with gestational hypertension, 64 with preeclampsia and 8 with severe preeclampsia, and 178 normotensive pregnant controls (51.4%). The HDP group had a significantly shorter median gestational age at admission [34.2 (30.6–37.4) weeks vs. 38.4 (37.2–39.6) weeks, *P* < 0.001], which was adjusted for in subsequent analyses. Baseline data are summarized in Table 1. The HDP group showed higher age, nulliparity rate, adverse pregnancy history rate, body weight, BMI, systolic blood pressure, diastolic blood pressure, fasting plasma glucose, triglyceride, low-density lipoprotein cholesterol, blood urea nitrogen, serum creatinine, aspartate transaminase, TyG index and TyG-BMI (all *P* < 0.05), along with lower gestational age, high-density lipoprotein cholesterol, albumin and platelet count (all *P* < 0.05). No intergroup differences were found in height, total cholesterol, alanine transaminase and hemoglobin (all *P* > 0.05).

**Table 1:** Comparison of baseline characteristics at admission between the two groups.

Variables	Control group (*n* = 178)	HDP group (*n* = 168)	*p*-value
General demographic data			
Age (years)	28.8 ± 4.2	30.6 ± 4.8	< 0.001
Gestational age at admission (weeks)	38.4 (37.2–39.6)	34.2 (30.6–37.4)	< 0.001
Primiparous, *n* (%)	92 (51.7)	112 (66.7)	0.005
Previous adverse pregnancy history, *n* (%)	22 (12.4)	36 (21.4)	0.022
Physical examination indicators			
Height (cm)	160.2 ± 4.8	159.4 ± 5.2	0.143
Admission weight (kg)	68.6 ± 8.4	76.8 ± 12.6	< 0.001
BMI (kg/m^2^)	26.8 ± 3.2	30.2 ± 4.6	< 0.001
BMI Grade, *n* (%)			< 0.001
< 24.0 kg/m^2^	32 (18.0)	12 (7.1)	
24.0–27.9 kg/m^2^	86 (48.3)	52 (31.0)	
28.0–31.9 kg/m^2^	48 (27.0)	62 (36.9)	
≥32.0 kg/m^2^	12 (6.7)	42 (25.0)	
Admission SBP (mmHg)	112 ± 8	152 ± 16	< 0.001
Admission DBP (mmHg)	72 ± 6	98 ± 12	< 0.001
Laboratory test indicators			
FPG (mmol/L)	4.52 ± 0.34	4.82 ± 0.46	< 0.001
TG (mmol/L)	2.86 (2.14–3.62)	3.48 (2.68–4.46)	< 0.001
TC (mmol/L)	6.12 ± 0.92	6.28 ± 1.06	0.140
HDL–C (mmol/L)	1.72 ± 0.34	1.56 ± 0.32	< 0.001
LDL–C (mmol/L)	3.26 ± 0.68	3.52 ± 0.76	0.001
BUN (μmol/L)	7.85 ± 2.56	9.28 ± 3.14	< 0.001
ALT (U/L)	13.8 (10.2–19.4)	15.2 (10.8–22.8)	0.116
AST (U/L)	18.4 (15.2–23.6)	21.6 (16.4–28.8)	0.002
Scr (μmol/L)	48.2 ± 8.6	54.6 ± 12.8	< 0.001
ALB (g/L)	34.2 ± 3.4	31.6 ± 4.2	< 0.001
Hb (g/L)	116.4 ± 12.6	118.2 ± 14.2	0.221
PLT ( × 10^9^/L)	218.6 ± 56.2	198.4 ± 64.8	0.003
Calculated indices			
TyG	8.82 ± 0.40	9.12 ± 0.46	< 0.001
TyG-BMI	196.8 ± 36.2	228.4 ± 44.6	< 0.001

BMI, Body mass index; SBP, Systolic blood pressure; DBP, Diastolic blood pressure; FPG, Fasting plasma glucose; TG, Triglyceride; TC, Total cholesterol; HDL-C, High-density lipoprotein cholesterol; LDL-C, Low-density lipoprotein cholesterol; UA, Uric acid; ALT, Alanine aminotransferase; AST, Aspartate aminotransferase; Scr, Serum creatinine; ALB, Albumin; Hb, Hemoglobin; PLT, Platelet count; TyG, Triglyceride glucose index; TyG-BMI, Triglyceride glucose-body mass index.

### 3.2 Correlation analysis between TyG-BMI and various parameters

The Spearman correlation analysis results are presented in Table 2. TyG-BMI showed significant positive correlations with BMI (*r* = 0.864, *P* < 0.001), TyG index (*r* = 0.756, *P* < 0.001), TG (*r* = 0.692, *P* < 0.001), FPG (*r* = 0.486, *P* < 0.001), UA (*r* = 0.342, *P* < 0.001), and LDL-C (*r* = 0.226, *P* < 0.001), while demonstrating significant negative correlations with HDL-C (*r* = −0.448, *P* < 0.001) and ALB (*r* = −0.248, *P* < 0.001). The strongest correlations were observed between TyG-BMI and BMI, as well as between TyG-BMI and TyG index, which aligns with the construction logic of this composite indicator.

**Table 2:** Spearman correlation analysis between TyG-BMI and each index.

Variables	Correlation coefficient (r)	*p*-value
BMI	0.864	< 0.001
TyG	0.756	< 0.001
TG	0.692	< 0.001
FPG	0.486	< 0.001
TC	0.238	< 0.001
HDL-C	−0.448	< 0.001
LDL-C	0.226	< 0.001
UA	0.342	< 0.001
Scr	0.186	< 0.001
ALB	−0.248	< 0.001
PLT	−0.142	0.008
Age	0.168	0.002
Gestational age at admission	−0.216	< 0.001

BMI, Body mass index; FPG, Fasting plasma glucose; TG, Triglyceride; TC, Total cholesterol; HDL-C, High-density lipoprotein cholesterol; LDL-C, Low-density lipoprotein cholesterol; UA, Uric acid; Scr, Serum creatinine; ALB, Albumin; PLT, Platelet count; TyG, Triglyceride glucose index; TyG-BMI, Triglyceride glucose-body mass index.

### 3.3 Multivariable logistic regression analysis

Logistic regression analyses were presented in Table 3. As a continuous variable, every 10-unit rise in TyG-BMI was linked to higher HDP risk, with OR values of 1.38 (95%CI: 1.24–1.54, *P* < 0.001) in the unadjusted model and 1.30 (95%CI: 1.16–1.46, *P* < 0.001) in the fully adjusted model. When stratified into quartiles with Q1 as reference, fully adjusted ORs were 1.76 (Q2, *P* = 0.132), 2.72 (Q3, *P* = 0.006) and 4.62 (Q4, *P* < 0.001), and the trend test reached statistical significance. In the fully adjusted model, per 1-unit increase in TyG index was associated with an OR of 2.42 (*P* < 0.001), and the highest quartile showed an OR of 3.28 relative to the lowest quartile (*P* = 0.002). For BMI, each 1-unit elevation corresponded to an OR of 1.18 (*P* < 0.001), and the OR between the highest and lowest quartiles was 3.04 (*P* = 0.004). Significant linear trends were observed for both indicators.

**Table 3:** Multivariate logistic regression analysis of the association between TyG-BMI, TyG index, BMI, and HDP.

Variables	Model 1 (OR:95%CI)	*p*-value	Model 2 (OR:95%CI)	*p*-value	Model 3 (OR:95%CI)	*p*-value
TyG–BMI						
Every 10 unit increase	1.38 (1.24–1.54)	< 0.001	1.34 (1.20–1.50)	< 0.001	1.30 (1.16–1.46)	< 0.001
Q1 ( ≤ 181.6)	1.00 (Reference)	—	1.00 (Reference)	—	1.00 (Reference)	—
Q2 (181.7–208.4)	2.02 (1.02–4.02)	0.044	1.86 (0.92–3.76)	0.084	1.76 (0.84–3.66)	0.132
Q3 (208.5–235.2)	3.18 (1.62–6.26)	< 0.001	2.92 (1.46–5.82)	0.002	2.72 (1.34–5.52)	0.006
Q4 (>235.2)	5.94 (3.02–11.72)	< 0.001	5.24 (2.60–10.56)	< 0.001	4.62 (2.16–9.90)	< 0.001
*p* trend	< 0.001		< 0.001		< 0.001	
TyG						
Every 1 unit increase	3.64 (2.46–5.40)	< 0.001	3.18 (2.10–4.82)	< 0.001	2.42 (1.52–3.86)	< 0.001
Q1 ( ≤ 8.62)	1.00 (Reference)	—	1.00 (Reference)	—	1.00 (Reference)	—
Q2 (8.63–8.94)	1.78 (0.88–3.58)	0.108	1.68 (0.82–3.42)	0.156	1.52 (0.72–3.18)	0.270
Q3 (8.95–9.26)	2.68 (1.36–5.30)	0.004	2.42 (1.20–4.86)	0.013	2.14 (1.04–4.42)	0.040
Q4 (>9.26)	4.46 (2.26–8.82)	< 0.001	3.82 (1.90–7.68)	< 0.001	3.28 (1.56–6.90)	0.002
*p* trend	< 0.001		< 0.001		< 0.001	
BMI						
Every 1 unit increase	1.30 (1.20–1.40)	< 0.001	1.24 (1.14–1.36)	< 0.001	1.18 (1.08–1.28)	< 0.001
Q1 ( ≤ 25.2)	1.00 (Reference)	—	1.00 (Reference)	—	1.00 (Reference)	—
Q2 (25.3–28.2)	1.68 (0.84–3.38)	0.146	1.54 (0.76–3.14)	0.232	1.40 (0.68–2.90)	0.364
Q3 (28.3–31.2)	2.86 (1.46–5.62)	0.002	2.52 (1.26–5.06)	0.009	2.22 (1.08–4.56)	0.030
Q4 (>31.2)	4.28 (2.14–8.56)	< 0.001	3.62 (1.78–7.36)	< 0.001	3.04 (1.44–6.42)	0.004
*p* trend	< 0.001		< 0.001		< 0.001	

Model 1: Unadjusted; Model 2: Adjusted for age, gestational age at admission, and number of pregnancies; Model 3 (fully adjusted), further adjusted for TC, HDL-C, UA, and previous adverse pregnancy history on the basis of Model 2. SBP and DBP were included as components of HDP diagnosis and were not included as covariates to avoid over-adjustment bias. To avoid multicollinearity, its component variables (TG, FPG, BMI) were not included in Model 3 when analyzing TyG-BMI; BMI was not included in covariates when analyzing TyG index; and TG and FPG were not included in covariates when analyzing BMI. SD, standard deviation.

### 3.4 Restricted cubic spline (RCS) analysis

**Figure 1:**
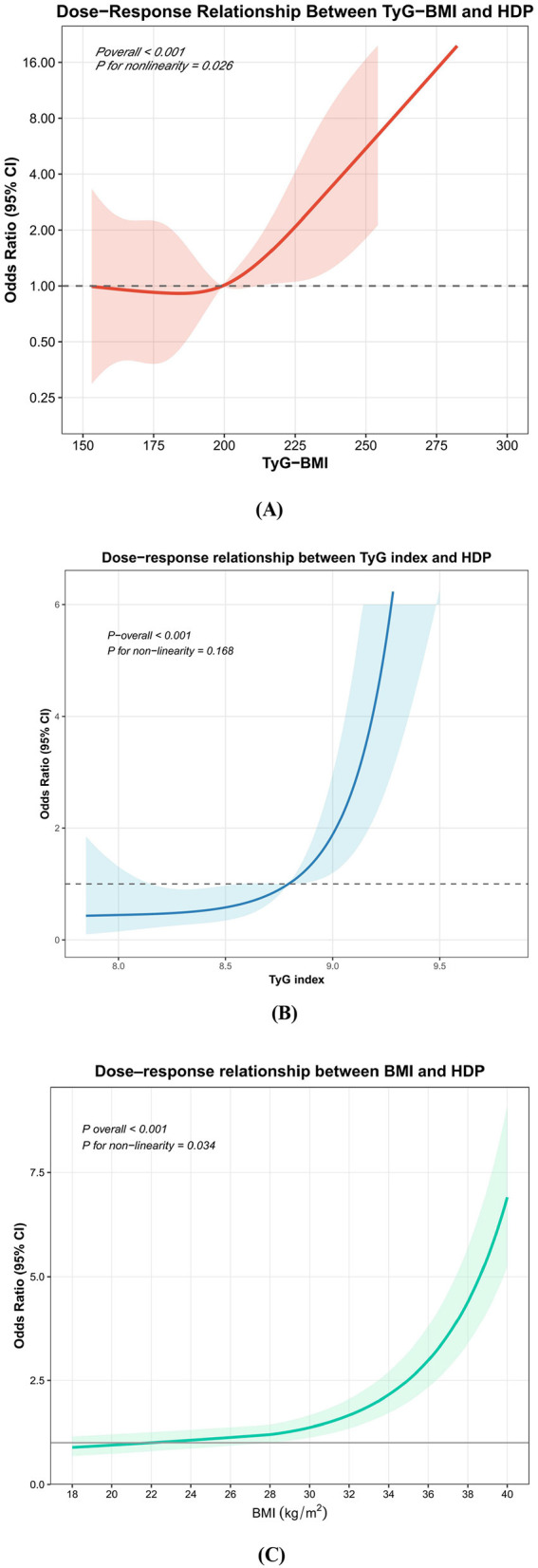
Restrictive cubic spline (RCS) analysis of TyG-BMI **(A)**, TyG index **(B)**, and BMI **(C)** vs. HDP. Solid lines represent point estimates of ORs and shaded areas represent 95% CIs. The median was taken as the reference point (OR = 1.0, horizontal dashed line). Nodes were set at the 5th, 25th, 50th, 75th, and 95th percentiles. Models were adjusted for age, gestational age at admission, number of pregnancies, TC, HDL-C, UA, and previous adverse pregnancy history.

Restricted cubic spline (RCS) analysis was used to explore the non-linear dose-response relationships between TyG-BMI, TyG index, BMI, and HDP (Figure 1). Figure 1A showed a significant overall (*P* overall < 0.001) and non-linear (*P* non-linearity = 0.026) association between TyG-BMI and HDP: HDP risk increased slowly when TyG-BMI < 200, but accelerated sharply beyond 200, indicating a potential threshold effect. Figure 1B revealed a significant overall association between TyG index and HDP (*P* overall < 0.001), but no significant non-linearity (*P* non-linearity = 0.168), suggesting a linear relationship. Figure 1C showed a significant overall (*P* overall < 0.001) and non-linear (*P* non-linearity = 0.034) association between BMI and HDP, with HDP risk accelerating markedly when BMI > 28 kg/m^2^.

### 3.5 ROC curve analysis

**Figure 2:**
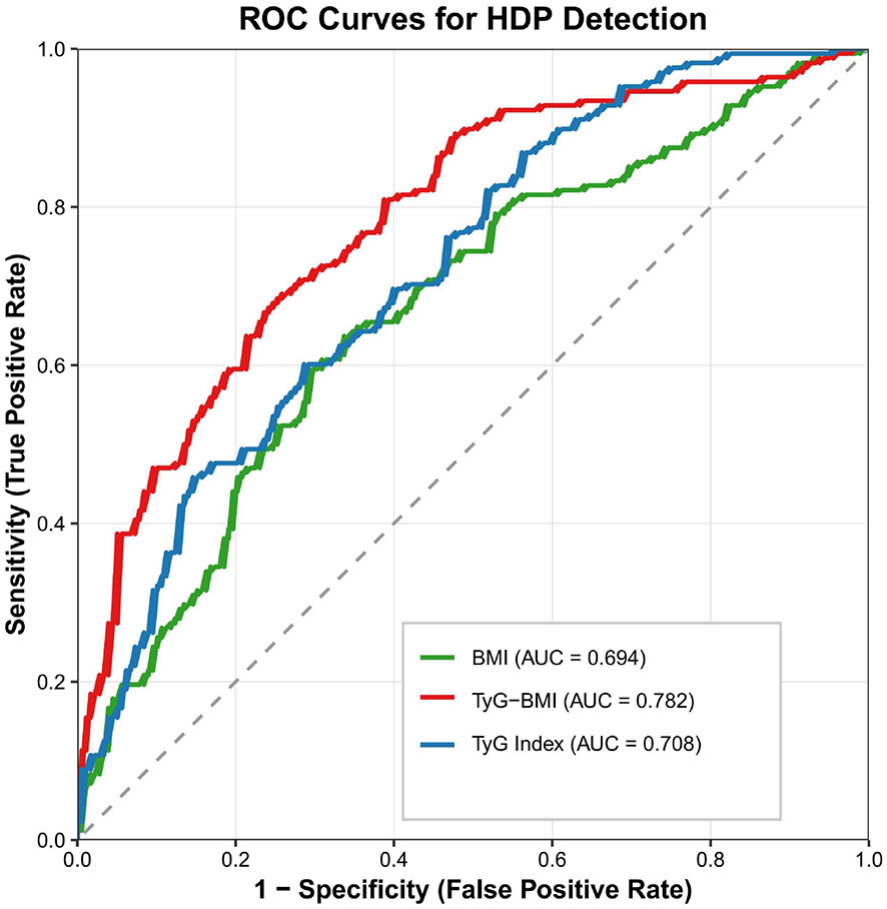
ROC curves of TyG-BMI, TyG index and BMI for discrimination of HDP. The red solid line is TyG-BMI (AUC = 0.782), the blue solid line is TyG index (AUC = 0.708), the green solid line is BMI (AUC = 0.694). The gray diagonal is the reference line (AUC = 0.5).

ROC curve analysis results are presented in Figure 2 and Table 4. The AUC for TyG-BMI in discriminating HDP was 0.782 (95%CI: 0.734–0.830), with an optimal cutoff value of 212.8, sensitivity of 72.6%, specificity of 72.5%, and Youden index of 0.451. The AUC for TyG index was 0.708 (95%CI: 0.656–0.760), with an optimal cutoff of 8.98, sensitivity of 67.3%, specificity of 64.6%, and Youden index of 0.319. The AUC for BMI was 0.694 (95%CI: 0.640–0.748), with an optimal cutoff of 28.2 kg/m^2^, sensitivity of 66.1%, specificity of 65.2%, and Youden index of 0.313. DeLong test results showed that the AUC of TyG-BMI was significantly higher than that of TyG index (Z = 2.862, *P* = 0.004) and BMI (Z = 3.412, *P* < 0.001).

**Table 4:** Comparison of ROC curve analysis for discrimination of HDP by each index.

Variables	AUC (95%CI)	Cut-off	Sensitivity (%)	Specificity (%)	Youden index	+LR	–LR	*p*-value (vs. TyG-BMI)
TyG-BMI	0.782 (0.734–0.830)	212.8	72.6	72.5	0.451	2.64	0.38	—
TyG	0.708 (0.656–0.760)	8.98	67.3	64.6	0.319	1.90	0.51	0.004
BMI	0.694 (0.640–0.748)	28.2	66.1	65.2	0.313	1.90	0.52	< 0.001

DeLong 's test was performed to compare the AUC of each index. *p* value (vs. TyG-BMI) is the AUC of each indicator compared with TyG-BMI AUC. + LR, positive likelihood ratio; – LR, negative likelihood ratio.

### 3.6 Subgroup analysis

**Figure 3:**
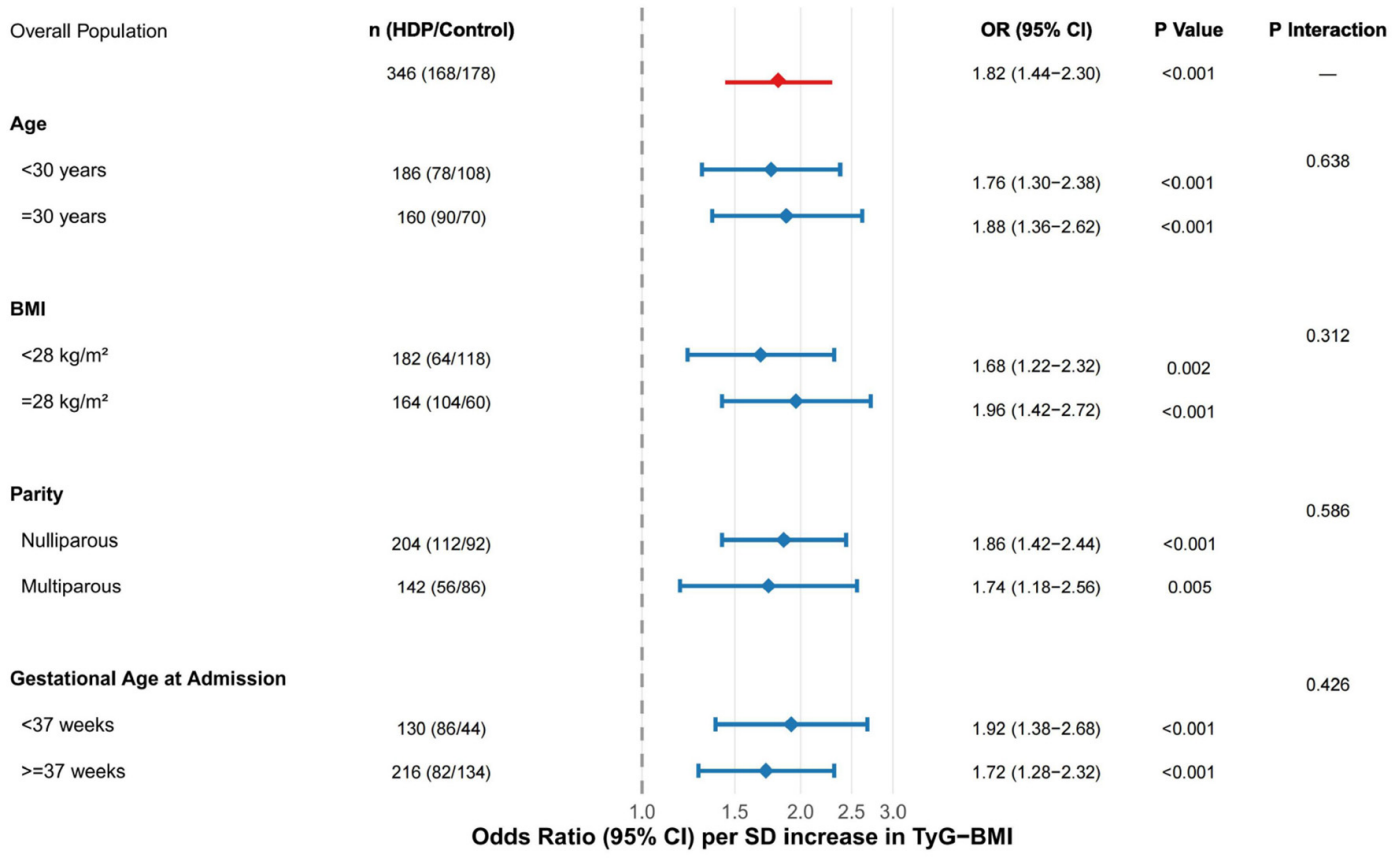
Forest plot for subgroup analysis of TyG-BMI association with HDP. Blocks represent point estimated ORs and horizontal lines represent 95% CIs. All interaction test *P*-values were > 0.05, suggesting no significant effect modification.

Subgroup analyses were performed according to age (< 30 years/≥30 years), BMI (< 28/≥28 kg/m^2^), parity (nulliparous/multiparous), and gestational age at admission (< 37 weeks/≥37 weeks). Results are shown in Table 5 and Figure 3. TyG-BMI maintained significant positive associations with HDP across all subgroups (all *P* < 0.05). All interaction tests were not statistically significant (all *P* interaction > 0.05), suggesting that the aforementioned factors do not significantly modify the association between TyG-BMI and HDP.

**Table 5:** Subgroup analysis of TyG-BMI association with HDP.

Subgroup	*n* (HDP/Control)	OR (95%CI)	*p*-value	*P* Interaction
Total population	346 (168/178)	1.82 (1.44–2.30)	< 0.001	—
Age				0.638
< 30	186 (78/108)	1.76 (1.30–2.38)	< 0.001	
≥30	160 (90/70)	1.88 (1.36–2.62)	< 0.001	
BMI				0.312
< 28	182 (64/118)	1.68 (1.22–2.32)	0.002	
≥28	164 (104/60)	1.96 (1.42–2.72)	< 0.001	
Number of pregnancies				0.586
Primiparous	204 (112/92)	1.86 (1.42–2.44)	< 0.001	
Multiparous	142 (56/86)	1.74 (1.18–2.56)	0.005	
Gestational age at admission				0.426
< 37	130 (86/44)	1.92 (1.38–2.68)	< 0.001	
≥37	216 (82/134)	1.72 (1.28–2.32)	< 0.001	

### 3.7 Sensitivity analysis

Multiple sensitivity analyses were performed to verify the robustness of the main results (Table 6): (1) Excluding the top and bottom 1% extreme values of TyG-BMI (*n* = 8), the significant association between TyG-BMI and HDP persisted (adjusted OR = 1.28, 95%CI: 1.14–1.44, *P* < 0.001). (2) Excluding 8 cases of severe preeclampsia (*n* = 338), the association remained consistent (adjusted OR = 1.28, 95%CI: 1.14–1.44, *P* < 0.001). (3) Subtype analysis showed a significant association in the gestational hypertension subgroup (*n* = 274, adjusted OR = 1.22, *P* = 0.002) and a stronger association in the preeclampsia subgroup (*n* = 242, adjusted OR = 1.40, *P* < 0.001). (4) Restricting analysis to gestational age ≥37 weeks (*n* = 216, adjusted OR = 1.26, *P* = 0.001) and 34–40 weeks (*n* = 264, adjusted OR = 1.28, *P* < 0.001) confirmed no fundamental impact of admission gestational age differences. (5) Propensity score matching (1:1, 126 matched pairs) showed a persistent significant association (adjusted OR = 1.26, *P* < 0.001). (6) Excluding BMI ≥ 32 kg/m^2^ (*n* = 292, adjusted OR = 1.24, *P* < 0.001) indicated the association was not solely driven by severe obesity. (7) Excluding FPG ≥ 5.1 mmol/L (*n* = 264, adjusted OR = 1.24, *P* = 0.002) confirmed the effect was not only due to elevated glucose.

**Table 6:** Summary of sensitivity analyses.

Analytical strategy	*n*	Adjusted OR (95%CI)†	*P*-value
Main analysis	346	1.30 (1.16–1.46)	< 0.001
Excluding extreme TyG-BMI (±1%)	338	1.28 (1.14–1.44)	< 0.001
Exclude severe preeclampsia	338	1.28 (1.14–1.44)	< 0.001
Gestational hypertension subgroup	274	1.22 (1.08–1.38)	0.002
Preeclampsia subgroup	250	1.40 (1.22–1.60)	< 0.001
Defined gestational age at admission ≥ 37 weeks	216	1.26 (1.10–1.44)	0.001
Defined gestational age at admission of 34–40 weeks	264	1.28 (1.12–1.46)	< 0.001
After propensity score matching	252	1.26 (1.10–1.44)	< 0.001
Excluded BMI ≥ 32 kg/m^2^	292	1.24 (1.10~1.42)	< 0.001
Excluded FPG ≥ 5.1 mmol/L	264	1.24 (1.08~1.42)	0.002

†OR is adjusted OR per 10 unit increase in TyG-BMI.Models were adjusted for age, gestational age at admission, number of pregnancies, TC, HDL-C, UA, and previous adverse pregnancy history.

## 4 Discussion

Based on clinical data from 346 hospitalized pregnant women at Fuyang Women and Children's Hospital, this study employed a case-control design to systematically investigate the association between TyG-BMI and HDP. The main findings are as follows: (1) TyG-BMI levels at admission were significantly higher in the HDP group compared to normal controls; (2) After adjusting for multiple confounding factors including age, gestational age at admission, parity, TC, HDL-C, UA, and adverse pregnancy history, TyG-BMI remained an independent factor associated with HDP, with each 10-unit increase in TyG-BMI associated with a 30% increased risk of HDP (adjusted OR = 1.30, 95%CI: 1.16–1.46), and a significant non-linear dose-response relationship between them; (3) The discriminative performance of TyG-BMI for HDP (AUC = 0.782) was significantly superior to TyG index (AUC = 0.708) and BMI (AUC = 0.694); (4) These associations remained consistent across different subgroups of age, BMI levels, parity, and gestational age at admission, and results were robust after multiple sensitivity analyses. These findings suggest that TyG-BMI, as a composite indicator integrating dual metabolic information of insulin resistance and obesity, can more comprehensively reflect the metabolic abnormalities in HDP patients compared to its individual components.

This study confirmed an independent positive correlation between elevated TyG-BMI and HDP, which is supported by solid pathophysiological evidence. The underlying biological mechanisms involve multiple interrelated pathways. Firstly, insulin resistance acts as a core link connecting metabolic disorders and the onset of HDP. Insulin sensitivity physiologically declines by approximately 50%−60% in late pregnancy to ensure sufficient glucose supply for fetal development (20). Nevertheless, such physiological insulin resistance is excessively aggravated into pathological insulin resistance in patients with HDP (7). A study conducted by Kaaja et al. (21) demonstrated that women with preeclampsia exhibited an additional 40% reduction in insulin sensitivity compared with normotensive pregnant women, and this difference remained statistically significant even after adjustment for BMI. Pathological insulin resistance facilitates the development of HDP via multiple mechanisms. Compensatory hyperinsulinemia can directly heighten sympathetic nerve excitability and accelerate renal tubular sodium reabsorption, thereby inducing fluid and sodium retention and subsequent blood pressure elevation. Moreover, insulin resistance impairs the phosphatidylinositol 3-kinase/endothelial nitric oxide synthase (PI3K/eNOS) signaling pathway, suppresses nitric oxide synthesis and release, and further triggers endothelial-dependent vasodilation dysfunction (22). In addition, it activates the mitogen-activated protein kinase (MAPK) pathway to upregulate endothelin-1 secretion and promote vascular smooth muscle cell proliferation, ultimately exacerbating vasoconstriction and vascular remodeling (23). As a validated surrogate marker for insulin resistance, increased TyG index directly reflects the activation status of the above-mentioned pathological processes. It must be emphasized that most of the above classic IR-mediated vascular injury signaling pathways are summarized based on general population metabolic disease researches, belonging to indirect mechanistic evidence; while the quantitative difference data of insulin sensitivity between pre-eclamptic pregnant women and normal pregnant women are direct pregnancy-specific research conclusions, which are more in line with the physiological state of gestational women.

Secondly, obesity contributes to the pathogenesis of HDP by mediating chronic inflammation and endothelial injury. Elevated body mass index (BMI), particularly excessive accumulation of visceral adipose tissue, represents a state of chronic low-grade inflammation. Adipose tissue secretes abundant pro-inflammatory cytokines such as tumor necrosis factor-α and interleukin-6, accompanied by reduced production of anti-inflammatory factors including adiponectin. This imbalance between pro-inflammatory and anti-inflammatory mediators further exacerbates insulin resistance, impairs vascular endothelial function and accelerates oxidative stress. A study by Ramsay et al. (24) verified that obese pregnant women present obvious vascular endothelial dysfunction and activated systemic inflammatory responses, with abnormal metabolic and vascular alterations emerging even before the onset of HDP. During pregnancy, maternal obesity further hampers trophoblast invasion and uterine spiral artery remodeling, aggravating shallow placental implantation and insufficient placental perfusion, which establishes a pathological foundation for the development of preeclampsia (25).Such inflammatory regulation and placental development interference effects are unique pathological manifestations in pregnant populations, which cannot be completely equated with the chronic inflammatory mechanism of simple obesity in non-pregnant crowds.

More importantly, insulin resistance and obesity do not merely exert independent effects, but interact to form a positive feedback loop. Obesity directly aggravates insulin resistance via imbalanced adipokine secretion and elevated free fatty acid levels. In turn, insulin resistance promotes lipogenesis and inhibits lipolysis through compensatory hyperinsulinemia, which further worsens obesity and ectopic fat deposition (26).As a combined indicator, TyG-BMI is calculated by multiplying the TyG index by BMI, which mathematically integrates the synergistic effects of insulin resistance and obesity. Consequently, it can more sensitively reflect the comprehensive impact of metabolic disorders on HDP than individual indicators alone. In the present study, TyG-BMI yielded an AUC value of 0.782 for distinguishing HDP, which was significantly higher than that of the TyG index (AUC = 0.708, *P* = 0.004) and BMI (AUC = 0.694, *P* < 0.001). These clinical findings further confirm the superior predictive performance achieved through such dimensional integration.

Consistent with the latest research results on glycolipid metabolic indicators in pregnant populations (12), single TyG index only focuses on glycolipid metabolism and cannot cover obesity-related chronic inflammatory risk information in pregnant women, leading to insufficient overall diagnostic accuracy for HDP; single BMI ignores the difference in intrinsic metabolic level among pregnant women with the same body mass, resulting in obvious screening bias in clinical risk stratification, which well explains the inherent performance defects of two single indicators confirmed in this study.

Restricted cubic spline (RCS) analysis in this study identified a significant non-linear dose-response relationship between TyG-BMI and HDP risk (*P* for non-linearity = 0.026). Specifically, HDP risk increased slowly when TyG-BMI was below approximately 200, while the slope of the risk curve rose sharply and presented an accelerated upward trend once TyG-BMI exceeded 200. This non-linear trend carries important pathophysiological implications, suggesting the existence of a metabolic load threshold effect. When the combined burden of insulin resistance and obesity surpasses the body's compensatory capacity, vascular endothelial damage and inflammatory responses may undergo qualitative aggravation, thereby leading to a disproportionate increase in HDP risk. This finding is consistent with the two-stage pathogenesis model of preeclampsia proposed by Redman and Sargent (27). On the basis of the first stage characterized by shallow placental implantation, excessive systemic maternal metabolic burden exceeding a certain threshold will markedly elevate the risk of developing clinical symptoms in the second stage.

Notably, BMI also exhibited a non-linear correlation with HDP risk (*P* for non-linearity = 0.034), with a marked acceleration in disease risk when BMI exceeded 28 kg/m^2^, which is basically in line with the diagnostic criteria for overweight and obesity among Chinese adults (28). In contrast, the association between TyG index and HDP risk tended to be linear (*P* for non-linearity = 0.168), indicating that within the value range of this study, the severity of metabolic insulin resistance was positively and evenly correlated with increased HDP risk. Compared with single indicators, TyG-BMI showed more prominent non-linear characteristics, which may reflect the exponentially amplified synergistic pathogenic effects of insulin resistance and obesity under high metabolic burden conditions.

ROC curve analysis revealed that TyG-BMI had an AUC value of 0.782 for identifying HDP, which was significantly higher than those of the TyG index (AUC = 0.708) and BMI (AUC = 0.694). This result is consistent with previous studies confirming the predictive value of TyG-BMI for cardiometabolic diseases in non-pregnant populations. Er et al. (28) firstly demonstrated that TyG-BMI was superior to the TyG index and other TyG-derived indicators in distinguishing insulin resistance among non-diabetic Korean adults. The present study further extended the diagnostic advantages of TyG-BMI from general adult populations to hospitalized obstetric patients, greatly broadening its clinical application scope.

From the perspective of information theory, the TyG index only includes fasting triglyceride and fasting plasma glucose to reflect glycolipid metabolism-related insulin resistance status, without evaluating individual obesity level. BMI merely reflects the ratio of body weight to height and lacks sufficient sensitivity to evaluate the degree of internal metabolic disorders. Both indicators have inherent information limitations. Pregnant women with elevated TyG index but normal BMI are prone to be classified as metabolically obese normal weight phenotype, while those with high BMI but low TyG index belong to metabolically healthy obese phenotype (13). The HDP risk of women with these two phenotypes is likely to be underestimated when assessed by single indicators. Through mathematical combination of two metabolic dimensions, TyG-BMI effectively compensates for the information defects of individual indicators.

Stratified analysis of HDP subtypes in sensitivity analysis showed that TyG-BMI had a stronger correlation with preeclampsia (including severe preeclampsia) (adjusted OR = 1.40) than with gestational hypertension (adjusted OR = 1.22), which has solid pathophysiological rationales. Systemic vascular endothelial dysfunction and excessive inflammatory reaction are the core pathological features of preeclampsia. During disease progression, increased soluble fms-like tyrosine kinase-1 (sFlt-1) and decreased placental growth factor (PlGF) secreted by the placenta cause severe vascular endothelial injury (29). Insulin resistance and obesity are critical metabolic risk factors driving endothelial damage and systemic inflammation, and their superimposed effects tend to exacerbate metabolic abnormalities in patients with preeclampsia. In comparison, gestational hypertension has more heterogeneous pathological mechanisms, and some cases are more closely associated with non-metabolic factors such as genetic susceptibility and mental stress (30). Accordingly, TyG-BMI serves as a more powerful biomarker for metabolism-related HDP, especially preeclampsia, which deserves further verification in future studies.

Subgroup analyses confirmed that the positive association between TyG-BMI and HDP remained stable across subgroups stratified by age, BMI, parity and admission gestational age. All interaction analyses showed no statistical significance (all *P* for interaction > 0.05), suggesting that the ability of TyG-BMI to reflect metabolic abnormalities in HDP is highly consistent among different pregnant populations. Of note, the effect size of TyG-BMI per standard deviation increment was numerically higher in the subgroup with BMI ≥ 28 kg/m^2^ (OR = 1.96) than in the subgroup with BMI < 28 kg/m^2^ (OR = 1.68), although no significant interaction was observed. This trend implies that the synergistic pathogenic effects of insulin resistance and obesity are more prominent in obese pregnant women, and the correlation between elevated TyG-BMI and HDP is more evident in this population (31). Nevertheless, such potential effect modification needs to be validated in studies with larger sample sizes due to the limited sample size of subgroups in this research.

Furthermore, multiple sensitivity analyses were performed to verify the robustness of our primary outcomes, and all analyses yielded consistent effect directions and statistical significance with the main results. Special attention was paid to eliminating the confounding effect caused by different admission gestational ages between groups. In clinical practice, patients with HDP are usually hospitalized earlier due to clinical symptoms, whereas healthy pregnant women are mostly admitted at term for delivery, leading to inevitable gestational age differences between the two groups. In this study, admission gestational age was already adjusted as a covariate in the main statistical model. Meanwhile, we conducted sensitivity analyses by restricting participants to women with admission gestational age ≥ 37 weeks (*n* = 216) and 34–40 weeks (*n* = 264). The results showed that the correlation direction and strength between TyG-BMI and HDP remained basically unchanged, with adjusted OR values of 1.26 and 1.28 respectively, confirming that gestational age difference did not substantially confound their association. In addition, the significant association still existed after excluding severely obese women with BMI ≥ 32 kg/m^2^ and participants with elevated fasting plasma glucose (FPG ≥ 5.1 mmol/L), indicating that this correlation was not driven merely by extreme obesity or hyperglycemia, but reflected the comprehensive effects of insulin resistance and obesity across a wide metabolic spectrum. Propensity score matching analysis further validated the stable association between TyG-BMI and HDP after balancing major confounding variables. The high consistency of all sensitivity analyses strongly supports the reliability and credibility of the conclusions drawn in this study.

This study has the following limitations. First, this study employed a single-center retrospective case-control design that cannot establish causal relationships between TyG-BMI and HDP, nor can it demonstrate prospective predictive value. This study can only reveal cross-sectional risk associations and discriminatory relationships at admission. Second, significant gestational age differences exist between groups (HDP group earlier), representing inherent case-control study characteristics since HDP patients are often admitted at earlier gestational ages due to medical needs. Although this study included gestational age at admission as a covariate adjustment in the main analysis and verified result consistency after limiting gestational age ranges in sensitivity analyses, different degrees of physiological metabolic changes during pregnancy between groups (such as physiological TG elevation in late pregnancy) due to gestational age differences may still retain some confounding effects. Third, BMI in this study represents admission values rather than pre-pregnancy BMI, with admission BMI including physiological weight gain throughout pregnancy and possible weight increases from edema in HDP patients, potentially overestimating baseline obesity levels.Fourth, the sample size was relatively limited (346 cases), with small sample sizes in some subgroup analyses (such as only 8 severe preeclampsia cases), potentially limiting testing power for interaction effects and rare subtypes. Fifth, all data originated from a single medical institution, with study population racial, regional, and socioeconomic characteristics potentially differing from other regions, requiring cautious interpretation of conclusion generalizability. Sixth, the case-control design determines that the TyG-BMI cutoff value (212.8) has only discriminative value rather than prospective predictive significance, and its clinical applicability requires further verification in prospective cohort studies.

In conclusion, this study confirmed an independent positive association between TyG-BMI and HDP, with a non-linear dose-response relationship between them. TyG-BMI's discriminative performance for HDP was significantly superior to TyG index and BMI, serving as a simple, economical, comprehensive indicator for identifying and assessing metabolic abnormalities in HDP patients. These findings require further verification in prospective, multicenter studies.

## Data Availability

The original contributions presented in the study are included in the article/supplementary material, further inquiries can be directed to the corresponding author.
